# Mitotic phosphorylation of the ULK complex regulates cell cycle progression

**DOI:** 10.1371/journal.pbio.3000718

**Published:** 2020-06-09

**Authors:** Akinori Yamasaki, Yui Jin, Yoshinori Ohsumi

**Affiliations:** 1 Cell Biology Center, Institute of Innovative Research, Tokyo Institute of Technology, Yokohama, Japan; 2 Tokyo Tech World Research Hub Initiative (WRHI), Institute of Innovative Research, Tokyo Institute of Technology, Yokohama, Japan

## Abstract

Autophagy is an intracellular degradation pathway targeting organelles and macromolecules, thereby regulating various cellular functions. Phosphorylation is a key posttranscriptional protein modification implicated in the regulation of biological function including autophagy. Under asynchronous conditions, autophagy activity is predominantly suppressed by mechanistic target of rapamycin (mTOR) kinase, but whether autophagy-related genes (ATG) proteins are phosphorylated differentially throughout the sequential phases of the cell cycle remains unclear. In this issue, Li and colleagues report that cyclin-dependent kinase 1 (CDK1) phosphorylates the ULK complex during mitosis. This phosphorylation induces autophagy and, surprisingly, is shown to drive cell cycle progression. This work reveals a yet-unappreciated role for autophagy in cell cycle progression and enhances our understanding of the specific phase-dependent autophagy regulation during cellular growth and proliferation.

## Abstract 2

This Primer explores how autophagy induction is regulated by the phosphorylation of ATG proteins, and highlights a recent study which reports that phosphorylation of ATG proteins by the cyclin-dependent kinase CDK1 drives cell cycle progression.

## Autophagy initiation and phosphorylation

Macroautophagy (hereafter autophagy) is a highly conserved, eukaryotic degradation pathway that isolates cellular materials into double-membrane vesicles, termed autophagosomes. Autophagosomes are ultimately degraded by fusion with the cell’s hydrolytic compartment, the lysosome (in mammalian cells) or vacuole (in yeast). Unlike proteasomal degradation, autophagy is capable of degrading entire organelles as well as macromolecules. Starvation for nutrients, such as amino acids or energy, triggers autophagy induction, which serves to overcome such stress by supplying nutrients derived from autophagic degradation. Autophagy is also induced in response to other stresses and occurs at a basal level in cells, contributing to the removal of deleterious cytosolic molecules and the maintenance of cellular homeostasis [1]. Many diseases, such as cancer, neurodegenerative diseases, infection, and inflammatory diseases have been linked to perturbed autophagy function [2].

In yeast, 18 autophagy-related genes (ATGs) are essential for autophagosome formation. The proteins encoded by these genes have been classified into 6 functional groups, with each group conserved between yeast and mammalian cells. These groups are as follows (with yeast terminology followed by mammalian counterparts in parentheses): the Atg1 (ULK) complex; the class III PI3K complex-I (PIK3C3-C1); the vesicle-localized membrane protein Atg9 (ATG9); 2 ubiquitin-like conjugation machineries; namely, the Atg8 (LC3/GABARAP) and Atg12 (ATG12) conjugation systems; and the Atg2-Atg18 (ATG2-WIPI) protein complex.

The yeast Atg1 complex is the most upstream component of the autophagy initiation process, receiving induction signals that regulate autophagy activity [3]. The proteins Atg1, Atg13, Atg11, Atg17, Atg29, and Atg31 act as components of the Atg1 complex. Atg1 itself is a serine/threonine kinase that phosphorylates Atg proteins upon autophagy induction. Atg17 is a primary scaffold protein that can form a subcomplex with Atg29 and Atg31 for bulk autophagy, which indiscriminately targets cytoplasmic components. Atg11 is also a scaffold protein that is essential for selective autophagy, which mediates the recognition of specific cargos through receptor proteins. Atg11 also plays a minor role in bulk autophagy. Meanwhile, Atg13 contains an intrinsically disordered region (IDR) and forms a subcomplex with Atg1.

The mammalian counterpart of the Atg1 complex, the ULK complex, is comprised of ULK1, ULK2, ATG13, ATG101, and FIP200. ULK1 and ATG13 are direct orthologs of Atg1 and Atg13, whereas ULK2 is a paralog of ULK1 that can compensate for the function of ULK1 [4]. There is no ATG101 ortholog in the yeast Atg1 complex, and the ULK complex also lacks counterparts for the yeast proteins Atg29 and Atg31. Evidence suggests that FIP200 is a functional homolog to the Atg1 complex scaffold subunits Atg11 and Atg17. Notably, Atg11, Atg17, and FIP200 are able to form a dimer [5–7].

Induction signals received by the ULK complex trigger autophagosome formation according to a sequential process employing the remaining ATG protein functional groups. First, ATG proteins nucleate, which is followed by the expansion of the autophagosome precursor, the isolation membrane. Finally, the autophagosome closes, capturing a portion of the cytoplasm. Phosphorylation of the ULK complex is critical for the regulation autophagy induction and the triggering of this autophagy machinery. Under nutrient-rich conditions, the ULK complex associates with the serine/threonine kinase mechanistic target of rapamycin (mTOR), resulting in mTOR-dependent ULK1/ULK2 and ATG13 phosphorylation [4] and the inactivation of ULK1 kinase activity, preventing autophagy induction. Upon starvation, the ULK complex dissociates from the inactivated mTOR, resulting in the dephosphorylation of ULK1/ULK2. This causes the activation of ULK1/ULK2 kinase activity and generates a positive feedback loop whereby ULK1/ULK2 undergoes extensive autophosphorylation. This activated ULK complex, together with PIK3C3-C1, serves as an autophagosome formation site adjacent to an endoplasmic reticulum (ER) microdomain called the omegasome that contains abundant phosphatidylinositol 3-phosphate (PI3P). The other ULK complex members (namely, ATG13, FIP200, and ATG101) then associate with the ULK complex to act as a scaffold recruiting downstream ATG protein functional groups.

Research in yeast has demonstrated that the Atg1 complex is a superassemblage that multiple Atg17-Atg29-Atg31 subcomplexes are linked with Atg1-Atg13 subcomplexes via 2 binding sites within Atg13 [8]. Furthermore, the activated Atg1 complex forms phase-separated droplets characterized by liquidity, thereby enabling dynamic and random molecular movement within the complex [9]. This liquidity is critical for the concentration and activation of Atg proteins by Atg1. Droplet formation is regulated by the phosphorylation state of the Atg13 IDR: Under nutrient-rich conditions, the Atg13 IDR is highly phosphorylated by TOR, and droplet formation is inhibited, whereas dephosphorylation of the IDR during starvation induces the droplet formation due to TOR inactivation and PP2C phosphatase recruitment into the Atg1 complex.

In mammals, it has been reported that other kinases are also able to phosphorylate ULK1. Adenosine monophosphate-activated protein kinase (AMPK) responds to energy depletion by phosphorylating Ser 317 and Ser 777 of ULK1 [10,11]. In contrast to phosphorylation by mTOR, AMPK phosphorylation promotes autophagy. AMPK phosphorylation is suppressed under nutrient-rich conditions because of mTOR phosphorylation of ULK1 preventing the interaction between ULK1 and AMPK.

Phosphorylation of substrates other than the ULK complex has also been shown to play important roles in modulating autophagy [1]. The class III PI3K complex produces PI3P, which is supplied to forming autophagosomes and regulates membrane traffic on the endosomal membrane [1]. There are 2 variants of PIK3C3 made up of common subunits (Vps34, Beclin 1, AMBRA1, and p115) and exclusive, variant-specific subunits (Atg14 or UVRAG). The variant containing ATG14 (PIK3C3-C1) is implicated in autophagy, whereas the UVRAG-containing variant (PIK3C3-C2) is involved in membrane trafficking. Both ATG14 and the common PIK3C3 component Beclin 1 can be phosphorylated [1,12]. Beclin 1 is a substrate of many kinases, such as ULK1, PGK1, AMPK, DAPK1/2/3, EGFR, Akt1, and CSNK1G2 [12]. In particular, Ser 30 phosphorylation of Beclin 1 by ULK1 is indispensable for PIK3C3-C1 activity. Numerous phosphorylation sites in the Beclin 1 C-terminal region promote UVRAG-containing PIK3C3 formation, thereby suppressing autophagy [12]. In addition, a transcription factor known as TFEB amplifies autophagy induction. Although TFEB is retained at the lysosome membrane under nutrient-rich conditions by mTOR kinase activity, inactivation of mTOR during starvation results in translocation of TFEB to the nucleus, where it enhances ATG protein expression.

Overall, it is clear that phosphorylation is intricately involved in the regulation of autophagy induction upon starvation. Phosphorylation allows for near-immediate and reversible responses to changes in the intracellular environment. However, little is known about how changes in phosphorylation observed throughout the phases of the cell cycle affect autophagy activity in proliferating cells.

## Phosphorylation as a dynamic mode of regulation during the cell cycle

Although cellular dynamics are a hallmark of the cell cycle in eukaryotic cells [13], research assessing the role of phosphorylation in autophagy regulation has almost exclusively been carried out under asynchronous conditions and remains unclarified for specific phases of the cell cycle. In order to proliferate, cells must synthesize a complete and accurate copy of the genome prior to cell division. Cells also need to amplify and partition their complement of cytoplasmic organelles prior to division [14,15]. The cell cycle is divided into 4 phases defined by the DNA content of the genome. The DNA synthesis phase is called the S phase, whereas the mitotic division phase is referred to as the M phase. There remaining 2 phases are gap phases between S and M known as the G1 and G2 phases. Each cycle progresses sequentially through G1, S, G2, and M phases. Throughout all phases, cyclin-dependent kinases (CDKs) function as the controller of the cell cycle to trigger different cell cycle events. CDK kinase activity is dependent on cyclin binding for its kinase activity; this cyclin-CDK complex drives the cell cycle by phosphorylating a range of target proteins that regulate pathways involved in cell cycle progression. In this way, phosphorylation and de-phosphorylation are regulatory switches that control cell cycle progression. Each stage of the cell cycle employs specific cyclin-CDK complexes. In mammalian cells, G1 cyclin-CDK4/6 is implicated in G1 phase progression, G1/S cyclin-CDK2 in the G1/S transition, S phase cyclin-CDK2 in the S phase, and M phase cyclin-CDK1 in both entry into and exit from the M phase [13,16]. It is well known that inhibition of M cyclin-CDK1 activity has dramatic effects: Assembly of the mitotic spindle, chromosome condensation, nuclear envelope breakdown, actin cytoskeleton rearrangement, and reorganization of the Golgi apparatus and endoplasmic reticulum are all perturbed [13,16–18]. In this issue, Li and colleagues skillfully use the M phase cyclin-CDK1 protein to unravel the relationship between autophagy and the cell cycle.

## Phosphorylation of the ATG proteins during mitosis

Although many substrates of cyclin-CDK complex have been identified by omics analyses, and it has been proposed that phosphorylation might have an impact on biological function [19], we are not fully understanding what effect cyclin-CDK complex–mediated phosphorylation has on autophagy. A limited number of studies have investigated autophagy regulation throughout the cell cycle [20], showing that although starvation or stress-induced autophagy preferentially occur during G1 and S phase, basal autophagy is detected at all phases [20]. It has been reported that the common PIK3C3 subunit Vps34 is phosphorylated by CDK1, thereby reducing the level of PI3P during mitosis [21]. In another study, PI3P production by Vps34 localizing to the midbody was reported to recruit Fab-1, YGL023, Vps27, and EEA1 (FYVE) domain-containing centrosomal protein (FYVE-CENT) for the completion of cytokinesis, the final step of mitosis [22]. The authors further reported that the impairment of PIK3C3 resulted in the arrest of cytokinesis. Disruption of autophagy also results in a cytokinesis defect due to accumulation of the small GTPase RHOA, a target of autophagy that causes genomic instability and is implicated in cancer progression [23]. In yeast, when cells are subjected to nutrient depletion, autophagy is critical for cell cycle progression. Autophagy maintains a sufficient amino acid pool for the progression of the cell cycle during starvation, suggesting that the impairment of autophagy causes G2/M arrest and aneuploidy [24,25]. These reports suggest that autophagy is required for proper mitosis, a function that is evolutionarily conserved throughout eukaryotes.

**Fig 1:**
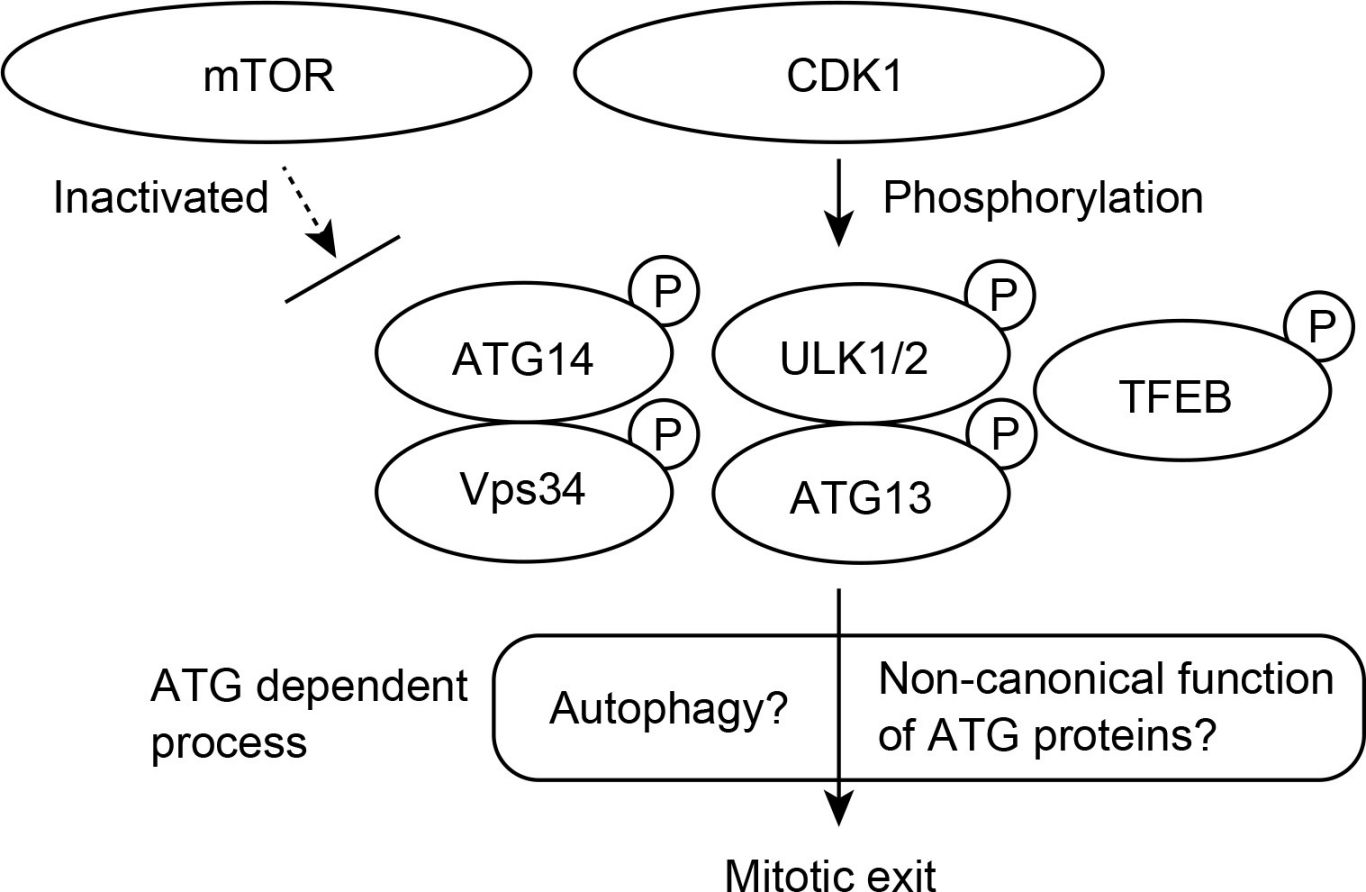
Phosphorylation of ATG proteins during mitosis. ATG proteins are phosphorylated by CDK1, not mTOR, during mitosis. Mitotic exit is subsequently mediated by ATG protein-dependent processes. ATG, autophagy-related genes; CDK1, cyclin-dependent kinase 1; mTOR, mechanistic target of rapamycin; TFEB,; ULK1/2,; Vps34.

Two papers (one by Li and colleagues in this issue and another recently published in *Molecular Cell* by Odle and colleagues) reveal that CDK1 phosphorylates the autophagy machinery [26,27] (Fig 1). In these works, both groups observe up-shifted bands of ULK1 and ATG13 in mitotic cell lysates. CDK1 was found to be responsible for this phosphorylation because the up-shifted bands were lost by CDK1 inactivation, even though mTOR is suppressed during mitosis. Furthermore, Odle and colleagues found that ATG14 and TFEB are also phosphorylated by CDK1. Both groups uncovered mitosis-specific phosphorylation sites in ULK1, ULK2 (Li and colleagues), ATG13, and ATG14 (Odle and colleagues) by mass spectrometric analyses (Fig 1). These data suggest that CDK1-dependent phosphorylation is a key means of autophagy regulation during the M phase of the cell cycle.

However, the authors propose different implications of CDK1-dependent phosphorylation for ATG proteins and mitotic autophagy. Odle and colleagues show that puncta numbers of ATG13 and WIPI, which are representative of autophagosome formation, did not increase with mTOR inhibition during mitosis. Thus, they conclude that autophagy is inhibited during mitosis. In contrast, Li and colleagues propose that the phosphorylation of ULK1 and ATG13 by CDK1 promotes autophagy during mitosis. Compared with wild-type cells, autophagy activity during mitosis was not promoted in ULK1/ATG13 double-knockout (KO) cells or in cells expressing nonphosphorylatable alanine substitution mutants of ULK1/ATG13. Moreover, Li and colleagues show that mitotic exit is impaired in ATG KO cells, providing evidence that mitotic autophagy is required for cell cycle progression. Based on these findings, the authors conclude that the ATG proteins are required for mitotic exit, although it is not yet clear whether this is achieved by mitotic autophagy or ATG-dependent noncanonical processes (Fig 1). Interestingly, autophagy appears to maintain optimal centrosome numbers through the degradation of the centriolar satellite component PCM1 [28]. Impairment of autophagy results in accumulation of abnormal centriolar satellites, with knock-on effects on microtubule assembly and cell cycle progression. Collectively, these findings suggest that the degradative function of autophagy is directly implicated in proper regulation of cell cycle progression.

Together, these works highlight the importance of phosphorylation of ATG proteins during mitosis. However, key questions remain to be answered, including whether the degradative function of mitotic autophagy is critical for cell cycle control, or whether the activities of ATG proteins themselves exert this affect. In the former case, substrates of autophagic degradation may regulate the cell cycle, with autophagy affording control over the availability of these proteins. In the latter case, ATG proteins themselves may act as regulators in a manner independent of the degradative function of autophagy. In addition, whether phase separation of the ULK complex is controlled by CDK1-mediated phosphorylation is another important question that must be addressed. Future studies will answer these questions and provide resolution to the question of how autophagy is involved in the regulation of the cell cycle.
